# Tuberculous pleural effusion with profuse mesothelial cell counts

**DOI:** 10.5339/qmj.2024.qitc.25

**Published:** 2024-03-25

**Authors:** Theeb Osama Sulaiman, Aasir M. Suliman, Abdullah Arshad, Mustafa A. Al-Tikrity, Irfan UI Haq

**Affiliations:** 1 Pulmonology Department, Hamad General Hospital, Hamad Medical Corporation, Doha, Qatar Email: TSulaiman@hamad.qa

**Keywords:** Tuberculosis, Mesothelial cells, Pleural effusion

## Introduction

Tuberculous pleural effusion (TPE) often leads to an exudative effusion characterized by a predominance of lymphocytes, with a scarcity of mesothelial cells. Studies confirm that the pleural fluid of tuberculosis (TB) patients rarely contains more than 5% mesothelial cells.^1,2^

## Case Presentation

A 33-year-old male presented with a three-month history of asthenia, anorexia, abdominal distension, and cough. On presentation, vital signs were stable except for mild tachycardia. Chest examination revealed reduced air entry on the lower right side. Abdominal examination showed ascites with positive shifting dullness and no lower limb edema. Laboratory findings showed mild anemia and a CRP of 48, otherwise unremarkable with a negative HIV test. Abdominal ultrasound showed mild ascites with no organomegaly. The chest X-ray showed mild blunting of the right costophrenic angle, and the chest ultrasound revealed a moderate right-sided pleural effusion.

**Figure 1.**
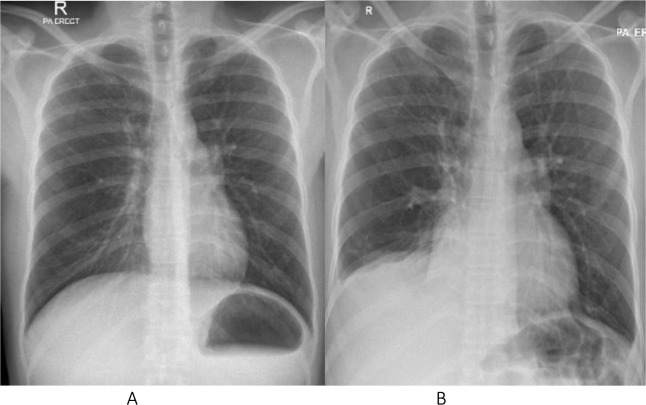
(A) Chest X-ray showed clear costophrenic angles compared to (B) – the image on admission, which showed blunting in the right costophrenic angle.

The result of ascitic fluid analysis showed a serum ascites albumin gradient (SAAG) of 2.3 g/l, with a lymphocyte percentage of 72%, a neutrophil percentage of 3%, and many mesothelial cells. Pleural fluid analysis revealed exudative fluid with a lymphocyte percentage of 26%, a neutrophil percentage of 2%, and a mesothelial cell percentage of 31%. The TB workup was negative for both ascitic fluid and pleural effusion. Medical thoracoscopy was not performed as the fluid was mainly located posteriorly. A laparoscopic peritoneal biopsy was performed, and histopathology showed necrotizing granulomatous inflammation consistent with TB. The patient was started on anti-TB medication and improved significantly clinically. A repeat chest X-ray 2 months later showed significant improvement (Figure 1).

## Conclusion

Higher mesothelial cell counts (HM), exceeding 5% of total cell counts in TPE, have rarely been described, especially in HIV patients. This is likely due to alterations in the immune response. HM in TPE in non-HIV-infected patients is extremely rare. Therefore, the presence of elevated mesothelial cells should not categorically preclude the consideration of TB pathology in these patients.

## Conflict of Interest

I have no conflict of interest.
